# Histopathological Evaluation of Human Placental Extract as a Direct Pulp-Capping Material in Dogs' Teeth

**DOI:** 10.1055/s-0044-1786841

**Published:** 2024-06-28

**Authors:** Rehab Khalil Safy, Mai Hamdy Ragab, Heba Bahgat Abdel-Maksoud

**Affiliations:** 1 Department of Conservative Dentistry, Faculty of Dentistry, Suez Canal University, Ismailia Governorate, Egypt; 2 Department of Endodontics, Faculty of Dentistry, Suez Canal University, Ismailia Governorate, Egypt; 3 Department of Conservative Dentistry, Faculty of Dentistry, Suez Canal University, Ismailia Governorate, Egypt; 4 Restorative Dentistry Department, King Abdulaziz University, Jeddah, Saudi Arabia

**Keywords:** histopathological evaluation, direct pulp capping, HPE, MTA

## Abstract

**Objective** The current research aimed to evaluate the histopathological pulpal alterations in dogs' teeth following direct pulp capping using either mineral trioxide aggregate (MTA) or human placenta extract (HPE).

**Materials and Methods** Forty-eight incisors with mature apices from four dogs were involved. The teeth were randomly allocated to three groups ( *n* = 16) based on the material utilized for direct pulp capping: MTA, HPE, and Teflon as the negative control group. All involved teeth were capped and restored at the same session. Each group was subsequently divided into two subgroups ( *n* = 8) based on the post treatment evaluation period: 2 and 4 weeks. The histopathologic changes in each specimen's pulp tissues, including pulp inflammation, hyperemia, necrosis, and dentin bridge development, were assessed. Then, all the data were statistically analyzed using chi-square, *t* -test, and one-way analysis of variance ( *p* < 0.05).

**Results** At 2 weeks, chronic inflammation was observed in 100% of MTA and 50% of HPE subgroups with a significant difference between them whereas the remaining 50% exhibited no inflammation. In contrast to Teflon that showed acute inflammation, remission of inflammation was time-dependent at both MTA and HPE subgroups as there was a substantial difference between the 2- and 4-weeks evaluation periods within the same group. However, throughout the initially observed 2 weeks, all subgroups exhibited essentially no complete calcified bridge; at 4 weeks, all MTA and HPE subgroups developed dentin bridge formation, with a significant difference between them regarding its thickness.

**Conclusion** HPE is a promising pulp-capping material inducing less intense chronic inflammation accompanied with thicker dentine bridge formation in comparison to MTA.

## Introduction

Vital pulp therapy research has been sparked by knowledge of the dentin–pulp complex's defensive response and a desire to develop minimally invasive endodontic treatment methods. 1 With the goal to preserve tooth vitality, dentin–pulp complex's regeneration is crucial. One of the essential pulp therapy techniques, direct pulp capping, aims to preserve the pulp's vitality through forming dentin bridges. 2 3 Pulp vitality is necessary for preservation of the normal physiological characteristics and structural integrity of teeth. Maintaining pulp's vitality after bacterial or iatrogenic injury is considered a serious dilemma. The challenging issue in contemporary restorative dentistry continues to be the establishment of biologically based, minimally invasive approaches designed to maintain pulp vitality. Vital pulp therapy through direct pulp capping was implemented as a trial to maintain the vitality and function of exposed dental pulp. 4 Through this technique, pulp exposure is directly capped with a biocompatible material, giving it the opportunity to form reparative dentin and preserve its vitality. 5 It is worth noting that proper sealing, reparative capabilities, as well as biocompatibility of the utilized capping material are considered cornerstones that influence the prognosis of the direct pulp-capping procedure. 6 As an effort to find a reliable bioactive material that seals dentin, promotes the development of an effective reparative dentin bridge, and activates cellular regeneration processes, huge number of materials and techniques have been investigated for direct pulp capping, but even so, some limitations have been recognized. 7

Calcium hydroxide was the most commonly used direct pulp-capping material for a long period but it had several drawbacks. 8 Therefore, mineral trioxide aggregate (MTA) was launched as a trial to overcome some of its limitations. 9 10 11 12 MTA nevertheless has numerous drawbacks, including challenging handling characteristics, prolonged setting times, elevated expenses, and a possibility of discoloration. 13 Consequently, search for more biomimetic approaches is motivated by the lack of an ideal pulp-capping product.

Recently, naturopathic remedies for many diseases through using placental tissues are receiving a considerable level of interest. 14 15 Although using of placenta for wound applications is well established currently, there has been a growing interest and implementation of innovative configurations of placental tissue for a wide range of therapeutic applications. 16 Placental tissue has consistently demonstrated therapeutic effects in clinical research; these effects have been linked to its content of collagens, fibronectins, proteoglycans, cytokines, and growth factors. As a result, it is considered a unique source of raw materials for the formation of novel structures for application in regenerative medicine. 17 Following the discovery of novel methods for placenta extraction and suspension preparation, considerable research on placenta as a therapeutic agent was conducted throughout the past few decades. In addition to being prosperous organ immunologically, the placenta has unique therapeutic properties such as analgesic, anti-inflammatory, immune regulating, and wound healing capabilities because of its valuable components. 18 Furthermore, studies demonstrated that animal placental extracts can exhibit therapeutic benefits comparable to those of human placenta. Consequently, many researchers are now convinced that human placenta extract (HPE) will completely change how modern medicine is practiced. Since HPE has an impact on healing of wounds, 14 15 it was hypothesized that capping with HPE could stimulate reparative dentin formation. Therefore, the current study's objective was to assess the pulpal reaction following pulp capping employing HPE in comparison to MTA in dog model. Null hypothesis that there was no difference between HPE and MTA's direct pulp-capping efficiency was tested in the current study.

## Materials and Methods

### Sample Size Calculations

G*Power version 3.1.9.2 was used to calculate the sample size. 19 20 With *α* level of 0.05 and *β* level of 0.05, the effect size *W* was 0.60 (big); power = 95%. The sample size was calculated to be 7 per each subgroup and raised to 8 to compensate for any laboratory preparation errors. Therefore, the estimated total sample size was 48 samples distributed into three groups according to the pulp-capping material ( *n* = 16) and each group divided into two subgroups according to the posttreatment evaluation periods; 2 and 4 weeks subgroups. 21

### Animals, Selection, and Preparation

The Research Ethics Committee, Faculty of Dentistry, Suez Canal University, Egypt (Ethical approval no. 634/2022), confirmed its approval to this study proposal. Throughout the course of the study, the authors committed to all institutional and international standards for the care and use of animals. The criteria for Animal Research Reporting In Vivo Experiments guidelines were followed. 22 The procedure was then performed at the Faculty of Veterinary Medicine, Suez Canal University, Egypt. Following sample size calculation, four healthy mature mongrel dogs weighing between 15 and 20 kg were recruited. To assure the histological development of their teeth pulp, all dogs chosen were between the ages of 1 and 2 years. Six maxillary and six mandibular incisors of each dog were included in the current study, summing up to 48 teeth. All included teeth were thoroughly examined to ensure that they are free of any cracks, caries, or pulp inflammation, which might adversely affect the healing process. 23 Every dog was examined, housed in an individual kennel, and watched over for 2 weeks. They were housed in clean, well-ventilated environments with a 12-hour light/dark cycle, and they had unrestricted availability of drink and food throughout the course of the study.

### Experimental Procedures

After a 12-hour fast, the dogs were premedicated through subcutaneous injection of 0.05 mg/kg atropine sulfate (Atropine sulfate 1%R; ADWIA, Egypt), in addition to intramuscularly injected 1 mg/kg xylazine HCl (Xylaject 2%R; ADWIA, Egypt), and 10 mg/kg ketamine HCl (KeiranR; EIMC Pharmaceuticals Co., Egypt). A dose of 25 mg/kg thiopental sodium (Thiopental sodium R; EIPICO, Egypt) was given intravenously to maintain anesthesia. Then, a rubber dam was utilized to isolate the operative field and all involved teeth were disinfected through a cotton pellet saturated with 2% chlorhexidine gluconate (JK Dental, A.R.E.).Using a #2 round carbide bur (SS White, Rio de Janeiro, Brazil), on the cervical third of the buccal surface, 0.5 to 1 mm above the gingival margin, and parallel to the cemento-enamel junction, class V cavities have been created. To avoid any thermal damage resulting from the procedure, the tooth and the cutting tools were properly irrigated with large quantities of sterile saline solution. 24 Pulp exposure of 1 mm diameter was performed in a standardized manner in a midpoint of the pulpal floor using a round bur. A sharp, sterile endodontic probe (DG16, Dental USA, Mc Henry, Illinois, United States) was utilized to expose the pulp tissue at the cavity floor. After pulp exposure and vitality confirmation, the bleeding was controlled by pressing cotton pellets moistened with buffered 0.5% NaOCl solution for 1 to 2 minutes to control bleeding. 25 Then, a proper isolation was performed by using cotton rolls.

**Fig. 1:**
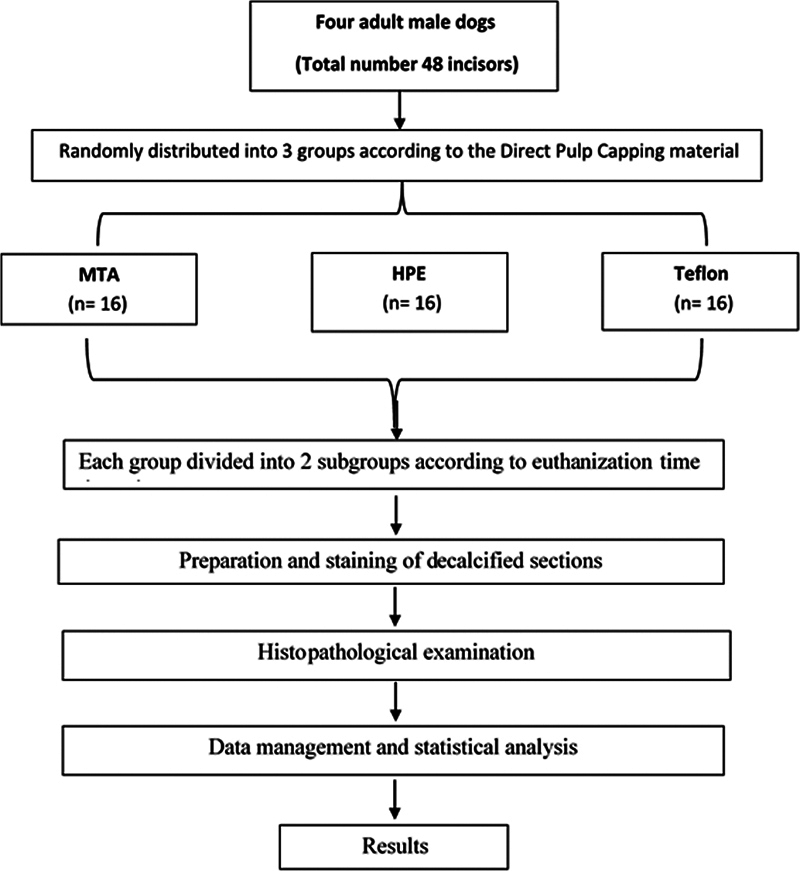
Flowchart of the current animal study.

A split mouth technique was followed in the current study where each pulp-capping material was represented in each dog by two maxillary and two mandibular incisors, where each pulp-capping material was equally presented in both right and left sides. The selected teeth of each dog were randomly distributed into three equal groups using Random Integer Set Generator, Randomness and Integrity Service Ltd ( http://www.random.org/ ). Grouping was performed depending on the material utilized for direct pulp capping including MTA, HPE, and Teflon (negative control group) ( Fig. 1 ). Capping of incisors in the MTA group was performed by manipulating the MTA pulp-capping material (Angelus, Londrina, PR, Brazil) according to the manufacturer's guidelines. Also, capping of the HPE group was accomplished through direct application of a thin layer of placenta extract gel (Placentrex extract Gel, Albert David, Kolkata, West Bengal, India) on the exposure site using a syringe. 26 For the teeth acting as the control group, a sterilized piece of Teflon (Teflon tape, Voco GmbH, Germany) was applied on the exposure site. 27 All cavities were immediately restored with a resin-modified glass ionomer cement (Fuji II LC, GC, Tokyo, Japan) and light-cured for 40 seconds. All dogs were given carprofen tablets (Rimadyl, Zoetis, United States), 4 mg/kg, once daily (by wrapping the tablet inside a tiny piece of meat) as a sedative for the following 5 days. 28 Two animals were randomly selected to be sacrificed in each of the posttreatment evaluation periods; 2 and 4 weeks subgroups. 29 A rapid injection of a general anesthetic solution (20 mL of thiopental sodium 5% solution) via the cephalic vein was used to help the dogs to euthanize. The jaws were separated to retrieve the experimental teeth.

### Histopathological Examination

The bone blocks were fixed in a 1:50 ratio of 10% buffered formalin solution. Specimens were decalcified in a 17% ethylenediaminetetraacetic acid solution (New Pharmchemical Co., Egypt) with a pH of 7. For around 120 days, the decalcifying solution was replaced on a daily basis, and decalcification was checked weekly. Following decalcification, the specimens were washed and maintained in water for 1 day to cease decalcification. The specimens were then dehydrated and fixed in paraffin blocks. For histopathologic examination, each block was sectioned in a buccolingual direction of 5-mm thickness, followed and stained with hematoxylin and eosin. 30 A digital camera mounted to a light microscope (Eclipse Ti-U, Nikon, Tokyo, Japan) was used to capture photomicrographs. All stained sections were examined by two experienced examiners at magnifications set to 200 and 400 in a blind manner. All photomicrographs were interpreted by the image analysis software Image J 1.41 (National Institutes of Health, Bethesda, Maryland, United States). The histopathologic features including pulp inflammation, hyperemia, necrosis, and dentin bridge formation were rated as the following 31 32 :

1. Inflammatory cell response
  (a) Type of inflammation:
    Score 0: without inflammation
    Score 1: acute
    Score 2: chronic
    Score 3: mixed
(b) Intensity (number of inflammatory cells in 100 μm ^2^ of the high-density area):
    Score 1: mild (0–30)
    Score 2: moderate (30–60)
    Score 3: severe (> 60)
  (c) Hyperemia (number of blood vessels):
    Score 1: 1–15
    Score 2: 15–30
    Score 3: > 30
  (d) Necrosis
    Score 0: without necrosis
    Score 1: signs of necrosis
2. Hard tissue formation (dentin bridge)
  Score 1: complete
  Score 2: little communication of capping material with pulp
  Score 3: lateral deposition of hard tissue on the cavity walls
  Score 4: absent
3. Hard tissue thickness
  Score 1: < 250
  Score 2: 150–249 μm
  Score 3: < 149 μm
  Score 4: absent

Each score was registered when the two observers scored an agreement of 0.81 (very good). When there was a disagreement, a consensus was reached. The development of hard tissue bridge was proposed as a sign for pulp-capping material efficiency. 33

### Statistical Analysis

Data coding and entering was performed through SPSS program version 28 (IBM Corp., Armonk, New York, United States). For quantitative variables, mean and standard deviation were used, and for categorical variables, frequencies (the number of cases) and relative frequencies (percentages) were used. Chi-square test was done to compare categorical data. Independent samples *t* -test was used to compare between 2 and 4 weeks in each group. Comparisons between groups were made using one-way analysis of variance with multiple comparisons Bonferroni post hoc for pairwise comparison in quantitative variables. A *p* -value of < 0.05 was regarded as statistically significant.

## Results

### Two Weeks' Observations

**Fig. 2:**
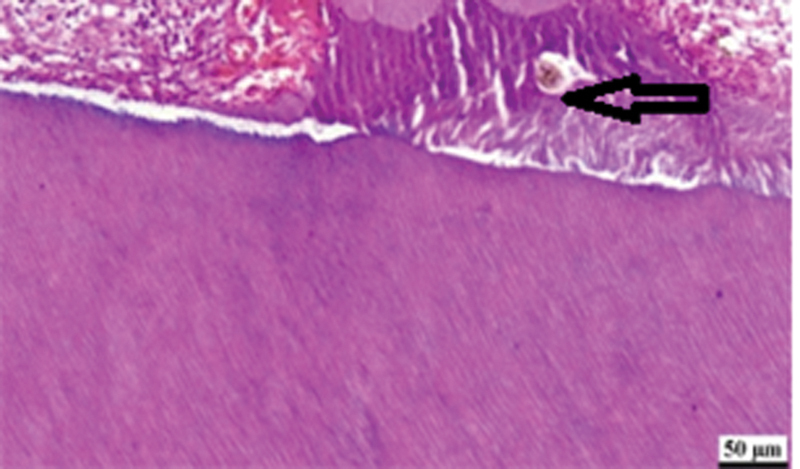
Photomicrograph of mineral trioxide aggregate subgroup at 2 weeks showing calcification (arrow), moderate inflammation, and congestion (hematoxylin and eosin [H&E] ×200).

**Fig. 3:**
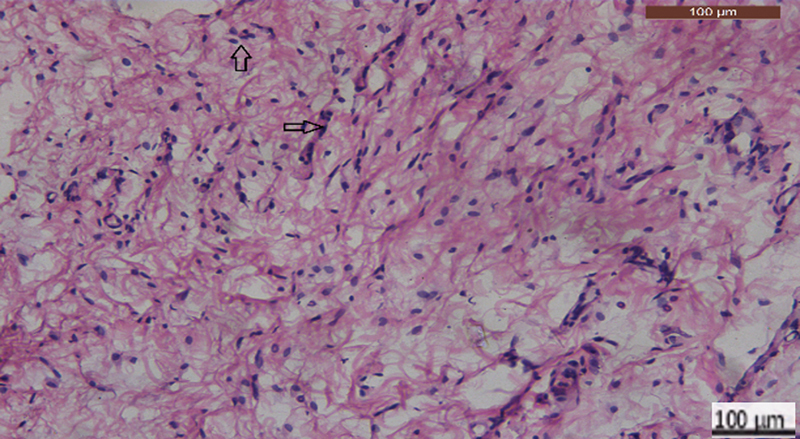
Photomicrograph of mineral trioxide aggregate subgroup at 2 weeks showing moderate inflammatory reaction (hematoxylin and eosin [H&E] ×400).

**Fig. 4:**
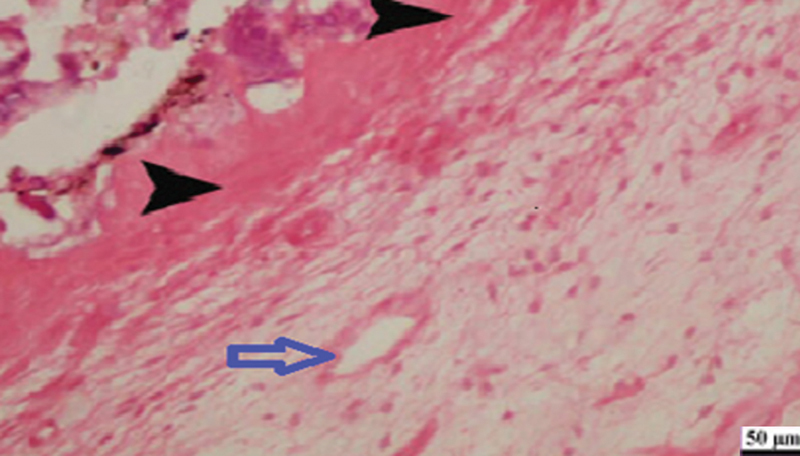
Photomicrograph of human placenta extract subgroup at 2 weeks showing dentin bridge formation (black arrow) and moderate inflammatory reaction (blue arrow) (hematoxylin and eosin [H&E] at 200× magnification).

**Fig. 5:**
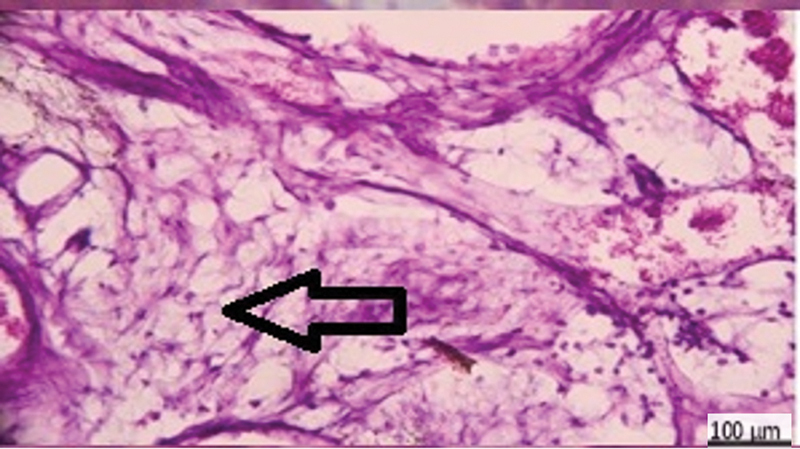
Photomicrograph of human placenta extract subgroup at 2 weeks showing moderate inflammatory reaction (arrow) (hematoxylin and eosin [H&E] ×400).

**Fig. 6:**
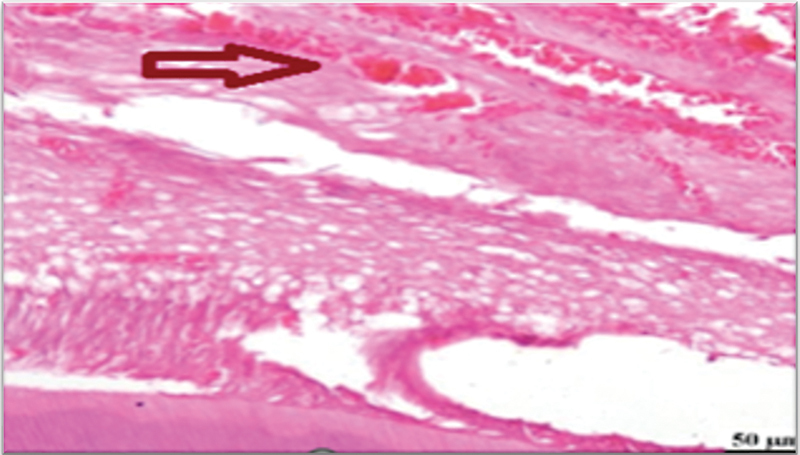
Photomicrograph of Teflon subgroup at 2 weeks showing extensive congestion (arrow), hemorrhage, and necrosis (hematoxylin and eosin [H&E] ×200).

**Fig. 7:**
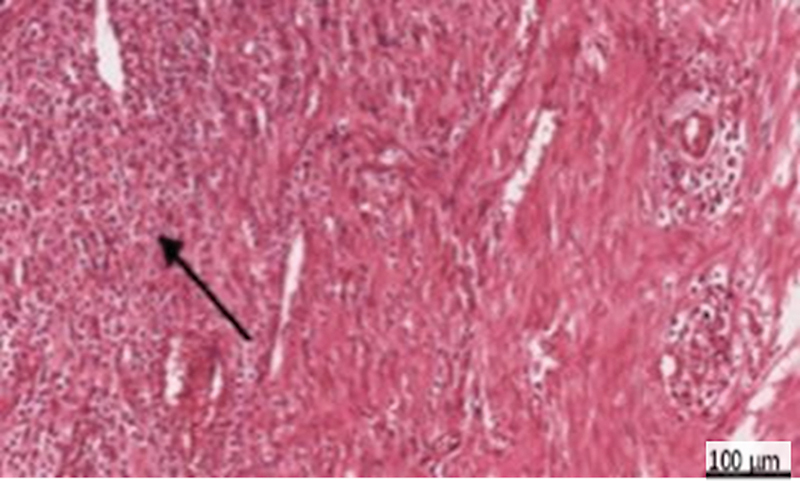
Photomicrograph of Teflon subgroup at 2 weeks showing proliferating fibrous tissue and inflammatory reaction (arrow) (hematoxylin and eosin [H&E] ×400).

Observing of data at Table 1 revealed that all teeth capped with MTA for 2 weeks, showed lateral deposition of hard tissue on the cavity walls ( Fig. 2 ), chronic inflammation of moderate intensity accompanied, and score 1 hyperemic reaction ( Fig. 3 ). In the same time, examining of 87.5% of pulp tissues capped with HPE showed also lateral deposition of hard tissue on the cavity walls without any significant difference with the MTA subgroup ( Fig. 4 ). Meanwhile, the remaining 12.5% of teeth showed little communication of the HPE capping material with the pulp tissue. In addition, it worth noting that 50% of teeth capped with HPE revealed chronic inflammation of moderate intensity ( Fig. 5 ), meanwhile the remaining 50% exhibited no inflammatory reaction. Also, score 2 hyperemia was noticed in all pulp tissues capped with HPE after 2 weeks with no significant difference with the MTA subgroup. No necrotic alterations were observed throughout the pulp tissues of all teeth capped with either MTA or HPE for 2 weeks ( Table 2 ). On the other hand, 87.5% pulp tissues capped with Teflon for 2 weeks showed acute inflammation of severe intensity and the remaining 12.5% revealed acute inflammation of moderate intensity. Most of Teflon-capped pulp tissues showed extensive congestion, hemorrhage, proliferating fibrous tissue, and inflammatory reaction ( Figs. 6 and 7 ). Evaluating the rate of blood vessel formation showed that highest rate was recorded in 62.5% of teeth capped with Teflon while the remaining 37.5% of teeth scored 2. Focal necrotic regions were somewhat dispersed in 37.5% Teflon-capped coronal pulp tissues along with substantial vascular dilatation in the nonnecrotic areas. A highly significant difference was recorded between Teflon and both MTA and HPE subgroups. Nevertheless, there was no evidence of a hard tissue bridge formation in 87.5% of the Teflon subgroup, 12.5% of these specimens had lateral deposition of hard tissue on the cavity walls. However, significantly higher thickness was recorded in the HPE subgroup in comparison to either the MTA or Teflon subgroups ( Table 3 ).

**Table 1:** Hard tissue bridge scores in different tested subgroups and significance of difference between them (chi-square test)

		MTA		HPE		Teflon		*p* -Values between groups
Variables	Scores	Count	%	Count	%	Count	%	
Hard tissue bridge after 2 wk	2	0	0	1	12.5	0	0	0.36 Ns
	3	8	100	7	87.5	1	12.5	0.068 Ns
	4	0	0	0	0	7	87.5	0.009 a
*p* -Value (MTA and HPE)		0.30 Ns						
Hard tissue bridge after 4 wk	1	7	87.5	8	100	0	0	0.022 a
	3	1	12.5	0	0	3	37.5	0.173 Ns
	4	0	0	0	0	5	62.5	0.0067 a
*p* -Value (MTA and HPE)		0.30 Ns						

Abbreviations: HPE, human placenta extract; MTA, mineral trioxide aggregate; Ns, not significant.

a Mean significant difference at *p* < 0.01.

**Table 2:** Number of frequency, percentages, and significance of signs of inflammation of all tested subgroups at both evaluation times

		MTA		HPE		Teflon		
Variables	Scores	Count	%	Count	%	Count	%	Between groups
Inflammation type after 2 wk	**0**	0	0	4	50	0	0	0.018 b
	**1**	0	0	0	0	8	100	0.0033 b
	**2**	8	100	4	50	0	0	0.0183 b
*p* -Value (MTA and HPE)		0.020 b						
Intensity after 2 wk	2	8	100	8	100	1	12.5	0.056 NS
	3	0	0	0	0	7	87.5	0.0009 b
*p* -Value (MTA and HPE		1.00 NS						
Hyperemia after 2 wk	1	8	100	0	0	0	0	0.0033 b
	2	0	0	8	100	3	37.5	0.0116 b
	3	0	0	0	0	5	62.5	0.0067 b
*p* -Value (MTA and HPE)		< 0.001 b						
Necrosis after 2 wk	0	8	100	8	100	5	62.5	0.65 NS
	1	0	0	0	0	3	37.5	0.0116 b
*p* -Value (MTA and HPE)		1.0 NS						
Inflammation type after 4 wk	0	8	100	8	100	0	0	0.0033 b
	1	0	0	0	0	8	100	0.0033 b
*p* -Value (MTA and HPE)		1.0 NS						
Intensity after 4 wk	1	5	62.5	8	0	0	0	0.0067 b
	2	3	37.5	0	0	1	12.5	0.173 NS
	3	0	0	0	0	7	87.5	0.009 b
*p* -Value (MTA and HPE)		0.054 NS						
Hyperemia after 4 weeks	1	5	62.5	8	100	0	0	0.023 b
	2	3	37.5	0	0	0	0	0.049 b
	3	0	0	0	0	8	100	0.0033 b
*p* -Value (MTA and HPE)		0.054 NS						
Necrosis after 4 wk	0	8	100	8	100	0	0	0.018 b
	1	0	0	0	0	8	100	0.0033 b
*p* -Value (MTA and HPE)		1.00 NS						

Abbreviations: HPE, human placenta extract; MTA, mineral trioxide aggregate; NS, not significant.

a Mean significant difference at *p* < 0.05.

b Mean significant difference at *p* < 0.01.

**Table 3:** Mean ± SD of histopathological criteria in tested materials

	Periods	MTA	HPE	Teflon	*p* -Value
Intensity	After 2 wk	42.33 ± 4.88 ^b^	37.83 ± 3.38 ^c^	65.66 ± 4.8 ^a^	< 0.001 d
	After 4 wk	25.39 ± 4.67 ^b^	5.7 ± 1.22 ^c^	73.79 ± 3.4 ^a^	< 0.001 d
*p* -Value		< 0.001 d	< 0.001 d	< 0.001 d	
Hyperemia	After 2 wk	17.83 ± 1.85 ^b^	17.08 ± 1.64 ^b^	33.39 ± 3.8 ^a^	< 0.001 d
	After 4 wk	7.89 ± 1.71 ^b^	4.35 ± 0.89 ^c^	37.67 ± 2 ^a^	< 0.001 d
*p* -Value		< 0.001 d	< 0.001 d	0.04 ^d^	
Dentin thickness	After 2 wk	32.0 ± 7.58 ^b^	202.63 ± 29.85 ^a^	20.0 ± 1.23 ^b^	< 0.001 d
	After 4 wk	216.75 ± 33.69 ^b^	493.88 ± 28.03 ^a^	15.67 ± 2.08 ^c^	< 0.001 d
*p* -Value		< 0.001 d	< 0.001 d	< 0.001 d	

Abbreviations: HPE, human placenta extract; MTA, mineral trioxide aggregate; SD, standard deviation.

Note: Mean significant differences between different groups at the same period (raw) at *p* < 0.01.

a indicating the highest mean value.

b indicating the mid level mean values.

c indicating the lowest mean value.

d Mean significant difference at *p* < 0.01.

### Four Weeks' Observations

The histopathological examination revealed that pulp tissue capped with either MTA or HPE for 4 weeks showed improved inflammatory response. Notably, the HPE subgroup experienced better recovery than the MTA subgroup, as all teeth capped with it exhibited mild or even absent inflammatory response with a significant difference with the MTA subgroup ( Table 3 ).

**Fig. 8:**
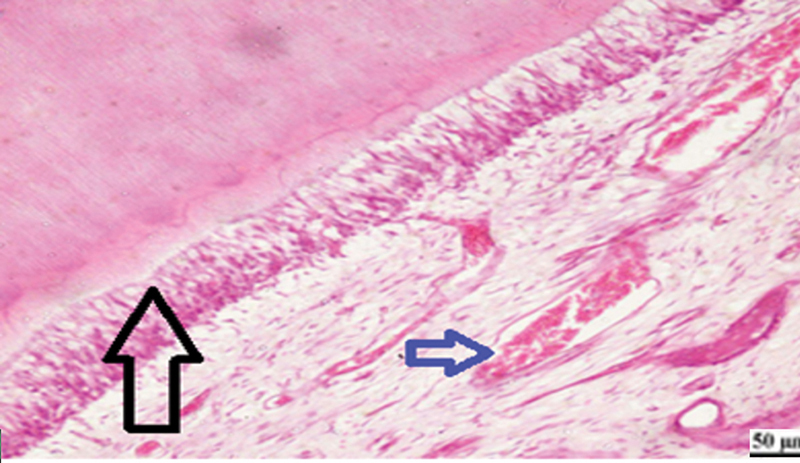
Photomicrograph of mineral trioxide aggregate subgroup at 4 weeks showing formation of dentin bridge (black arrow), pulp tissues showing dilated blood vessels (blue arrow) (hematoxylin and eosin [H&E] ×200).

**Fig. 9:**
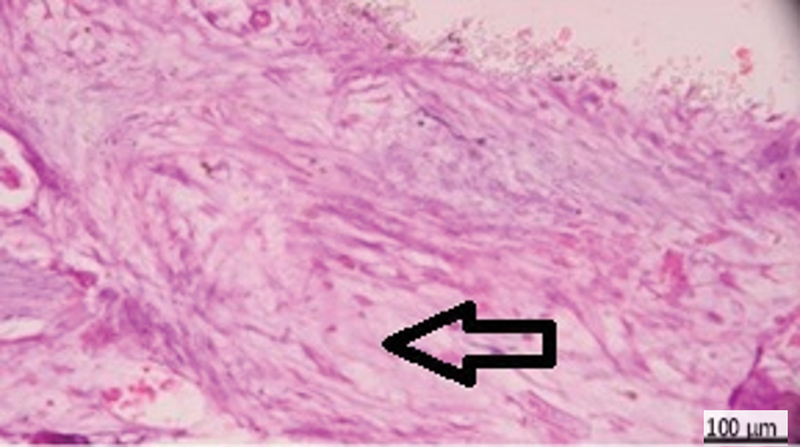
Photomicrograph of mineral trioxide aggregate subgroup at 4 weeks showing mild inflammation and minimal hyperemic reaction (arrow) (hematoxylin and eosin [H&E] ×400).

**Fig. 10:**
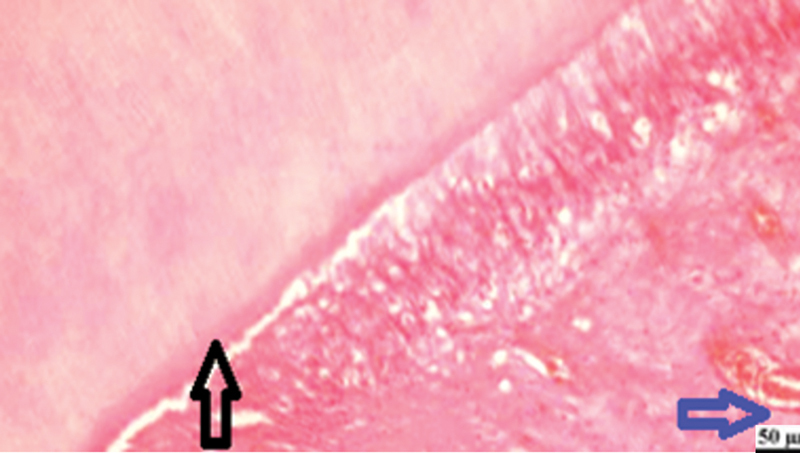
Photomicrograph of human placenta extract subgroup at 4 weeks showing minimal hyperemic reaction (blue arrow) and continuous dentin bridge formation (black arrow) (hematoxylin and eosin [H&E] at ×200).

**Fig. 11:**
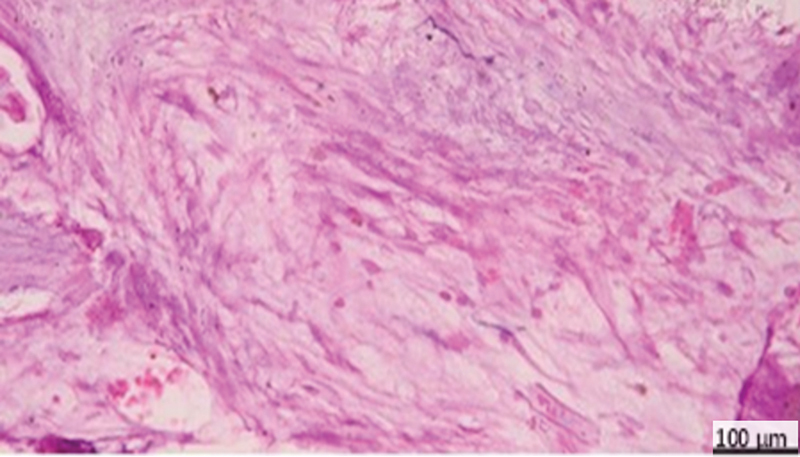
Photomicrograph of human placenta extract subgroup at 4 weeks showing absence of inflammation (hematoxylin and eosin [H&E] ×400).

**Fig. 12:**
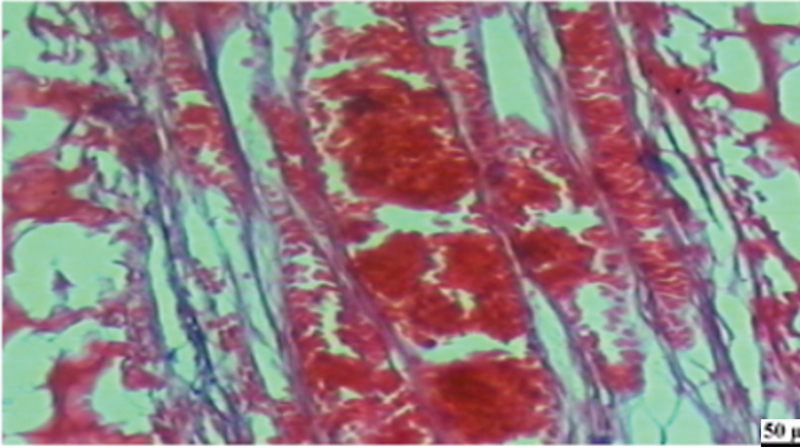
Photomicrograph of Teflon subgroup at 4 weeks showing severe inflammatory reaction, healing with connective tissue, and absence of dentin bridge (hematoxylin and eosin [H&E] ×200).

**Fig. 13:**
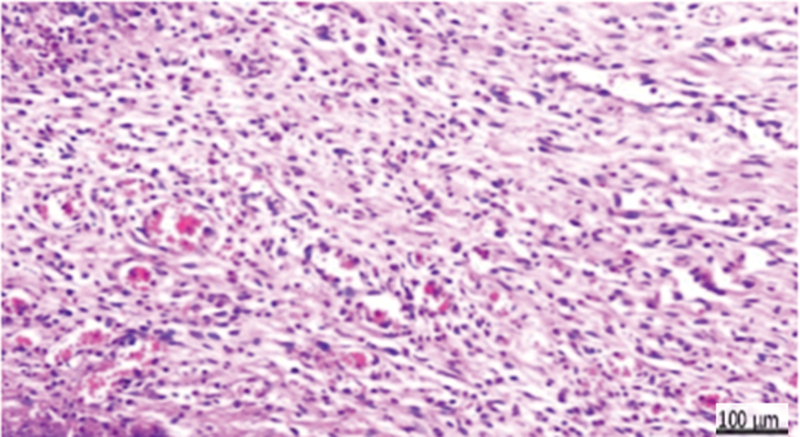
Photomicrograph of Teflon subgroup at 4 weeks showing severe inflammatory reaction with intense lymphocytic inflammatory infiltrate (arrow) (hematoxylin and eosin [H&E] ×400).

Evaluation of the hard tissue formation showed that the pulp tissues of 87.5% of teeth capped with MTA exhibited complete newly formed dentin bridge (score 1, Fig. 8 ) and lateral deposition of hard tissue on the cavity walls was observed in 12.5% of MTA cases (score 3). In terms of analyzing the inflammation intensity, 62.5% of the MTA subgroup displayed mild inflammation ( Fig. 9 ), whereas 37.5% displayed moderate one ( Table 2 ). Examining of pulp tissue capped with HPE revealed that all teeth capped with it exhibited newly formed dentin tissue bridge that cap the exposure site completely ( Figs. 10 and 11 ) with no significant difference with the MTA subgroup ( Table 1 ). According to data in Table 3 , the newly formed dentin bridge produced by HPE was of significantly higher thickness than that recorded for the MTA subgroup. On the other side, all teeth capped with Teflon for 4 weeks exhibited an exaggerated moderate to severe acute inflammatory response, focal necrotic regions with substantial pulp congestion throughout the coronal pulp tissue ( Figs. 12 and 13 ).

## Discussion

Direct pulp capping is a vital pulp therapy that attempts to preserve the viability of the pulpal tissues via protecting the pulpal system from bacterial invasion and so boosting its potential for repair. Effective direct pulp capping eliminates the need for more complicated or invasive procedures like extraction or root canal treatments. 34 The quest to find novel materials whose properties can be harnessed for dental regeneration due to lack of an optimal pulp-capping material is the primary driving force behind the investigation of further pulp capping strategies. Based on the biologically based wound-healing strategies of HPE, 35 36 it was evaluated in the current study as it may be of value in the future of vital pulp therapy targeting pulp regeneration processes.

Dogs were chosen as the study's animal model in the current study due to similarity of reparative dentin formation to that in humans, however, occurs at a faster rate. The dog also has an acceptable pulp dimension for histopathologic examination and a sufficient number of incisors for comparing multiple pulp-capping materials in the same dog, reducing the number of sacrificed animals in the study. 37 Split mouth technique was performed in the current study to eliminate the interindividual variability for the evaluation of the treatment effect. As a way to facilitate material handling along with offering protection from occlusal stresses, class V cavities were constructed. The pulps were exposed by mechanically perforating the cavity floor using a probe. This method is recommended as it uniformly exposes the pulp while protecting it from serious damage. 38 Although using this approach might cause dentin fragments to be pushed into the pulp, this has not been proved to cause an inflammatory pulpal reaction. 39

Teflon, a chemically inert substance, was utilized in the current study's uncapped pulp as a negative control group to distinguish the difference between the capping materials' stimulating effect and pulp self-reparative reaction. 40 Examination of the pulp tissue at 2 and 4 weeks revealed that pulp capping with Teflon tape frequently resulted in tissue damage, massive inflammatory reaction, and a lack of dentin bridge development at the exposed site. This was assumed to be due to Teflon's inability to encourage pulpal healing or stimulate the development of a continuous dentin bridge at the exposed areas. Nevertheless, some of the control specimens at week 4 exhibited the lateral deposition of hard tissue development with a thin thickness, which is in accordance with other studies. 41 42 This result could be attributed to the ability of pulp to repair and generate dentin bridges in the absence of bacterial microleakage.

A readymade single-paste HPE was utilized as a direct pulp-capping material in the current study. In contrast with MTA, this paste is characterized by better handling properties, shorter setting time, and lower cost. Despite the fact that initial pulpal inflammation is essential for healing and regeneration, 43 accelerating the resolution of the inflammatory response would benefit the process of dental regeneration. The biocompatibility of both tested materials when utilized to cover mechanically exposed tooth pulp was proven in the present study, as both MTA and HPE specimens displayed subsided inflammation and increased formation of continuous hard tissue bridges by time. Higher resolution of inflammatory reaction of HPE specimens by time in comparison to MTA could be attributed to its immunologically privileged role, 44 which played on both cell medicated and humoral through raising of total lymphocytes count as long as immunoglobulin (Ig) G and IgM levels raised significantly.

Furthermore, after 4 weeks, a considerably thicker layer of dentin bridge developed at the exposed area along with noticeably diminished inflammation, suggesting the more favorable results of HPE over MTA. The recognition that HPE is a unique reservoir and a genuinely marvelous source of multiple growth factors 45 can be leveraged to clarify the reason why HPE was effective in this study as a pulp-capping material. This result could be attributed to its unique bone morphogenetic proteins (BMPs) which are a specific family of growth factors. This family of growth factors had proved its essential role in dentin formation 46 and regeneration. 47 48 According to these reports, the differentiated odontoblast continues to produce dentin as a result of BMP signaling. 46 Specifically, BMP2 plays a key role as a regulator of the odontoblastic differentiation process and supports forming mineralized nodules. 49 Consequently, in a sequential cascade, recombinant human BMP2 stimulate pulp stem cells to differentiate into odontoblasts, increase their alkaline phosphatase activity and accelerate dentin sialophosphoprotein gene expression accompanied with improved hard tissue formation. 50

Additionally, HPE plays an important role in stimulating the release of substantial amounts of transforming growth factor- β1 18 that boost odontoblasts activity and encourage the reparative dentinogenesis. 51

It is worth noting that the current results were obtained under the limitations of the present study, which included a limited sample size and short evaluation periods. Additionally, the results of the current study were obtained from traumatic exposure of healthy pulp tissue. Therefore, any comparison to an inflamed pulp should be done with caution. Consequently, to validate the current results and assess HPE's clinical relevance and cost-effectiveness as a direct pulp-capping material, more extensive, carefully planned clinical research with larger sample sizes in various clinical settings is required. Also proving its effectiveness in maintaining pulp vitality, through evaluating its compatibility, the quality of formed hard tissue bridge, possibility of discoloration, and bond strengths with the overlying permanent restorative materials should be a topic of discussion in the upcoming studies.

## Conclusion

Under the limitations of the current study HPE could be a promising avenue for direct pulp capping owing to stimulation of more dentin bridge formation with less intense inflammatory response in comparison to MTA.
